# Effects of a lifestyle program in subjects with Impaired Fasting Glucose, a pragmatic cluster-randomized controlled trial

**DOI:** 10.1186/s12875-015-0394-7

**Published:** 2015-12-22

**Authors:** Arlette E. Hesselink, Guy E. H. Rutten, Sander M. Slootmaker, Inge de Weerdt, Lieke G.M. Raaijmakers, Ruud Jonkers, Marloes K. Martens, Henk J. G. Bilo

**Affiliations:** ResCon, Research & Consultancy, Kennemerplein 7, 2011 MH, Haarlem, The Netherlands; Julius Center for Health Sciences and Primary Care, University Medical Center Utrecht, Utrecht, The Netherlands; Netherlands Diabetes Federation, Amersfoort, The Netherlands; Department of Health Promotion, NUTRIM School for Nutrition, Toxicology and Metabolism, Maastricht University Medical Centre, Maastricht, The Netherlands; Diabetes Centre and Department of Internal Medicine, Isala Clinics, Zwolle; and, University Medical Centre Groningen, Groningen, The Netherlands

**Keywords:** Prevention, Nurse-led lifestyle intervention, Impaired fasting glucose, Primary care, Family practice, Health services, Randomized controlled trial

## Abstract

**Background:**

The worldwide epidemic of type 2 diabetes (T2DM) underlines the need for diabetes prevention strategies. In this study the feasibility and effectiveness of a nurse led lifestyle program for subjects with impaired fasting glucose (IFG) is assessed.

**Methods:**

A cluster randomized clinical trial in 26 primary care practices in the Netherlands included 366 participants older than 45 years with newly diagnosed IFG and motivated to change their lifestyle (intervention group, *n* = 197; usual care group, *n* = 169). The one-year intervention, consisting of four to five individual nurse-led consultations, was directed at improving physical activity and dietary habits. The primary outcome measure was body mass index (BMI). Linear and logistic multilevel analyses and a process evaluation were performed.

**Results:**

Both groups showed small reductions in BMI at 1 and 2 years, but differences between groups were not significant. At both 1 and 2-year follow-up the number of participants physically active for at least 30 minutes at least five days a week was significantly improved in the intervention group compared to the usual care group (intervention group vs. usual care group: OR_1year_ = 3.53; 95 % CI = 1.69-7.37 and OR_2years_ = 1.97; 95 % CI = 1.22-3.20, respectively). The total drop-out rate was 24 %. Process evaluation revealed that participants in the intervention group received fewer consultations than advised, while some practice nurses and participants considered the RM protocol too intensive.

**Conclusions:**

This relatively simple lifestyle program in subjects with IFG resulted in a significant improvement in reported physical activity, but not in BMI. Despite its simplicity, some participants still considered the intervention too intensive. This viewpoint could be related to poor motivation and an absence of disease burden due to IFG, such that participants do not feel a need for behavioural change. Although the intervention provided some benefit, its wider use cannot be advised.

**Trial registration:**

Current Controlled Trials ISRCTN41209683, date of registration 16/10/2013h  .

## Background

The worldwide epidemic of type 2 diabetes (T2DM) underlines the need for diabetes prevention strategies that can be implemented in daily practice in a cost-effective and efficient manner. Impaired fasting glucose (IFG) is a risk factor for T2DM [1]. Lifestyle changes in physical activity and dietary habits in subjects with IFG appear to be effective in preventing the development of diabetes, and are particularly important because individuals with IFG also show increased cardiovascular risk [2].

For subjects with IFG, Dutch guidelines for primary care physicians advise a yearly check-up for diabetes-related symptoms and blood glucose levels. Cardiovascular risk factor analysis is also advised, followed by appropriate treatment in case of increased risk [3]. Addressing IFG requires the support and coaching of diagnosed individuals in a systematic manner, preferably based on well-proven interventions. Lifestyle interventions that focus on improving both physical activity and diet can be (cost)effective [4–7]. A Dutch study demonstrated that people at risk for T2DM and/or cardiovascular disease can be motivated to change their lifestyle, leading to a sustained improvement in glucose tolerance [8]. However, another study concluded that annual consultation with a practice nurse provides insufficient support to individuals attempting to maintain lifestyle changes aimed at countering increased risk for diabetes or cardiovascular disease; a more intensive approach was deemed necessary [9].

The importance of intensive support was highlighted in two reviews that studied the feasibility of diabetes prevention programs. Both found that almost all effective diabetes prevention programs were intensive, and required both considerable manpower and rigorous supervision [10, 11]. However, due to financial and staffing restraints, intensive interventions are rarely feasible or sustainable in daily practice. Practicality requires the development of less intensive and less expensive (but still effective) interventions that can be more closely tailored to the needs of patients. It remains an open question whether effective, low intensity programs can be developed. Additionally, a recent meta-analysis reported that lifestyle interventions adhering more closely to recommended lifestyle modification guidelines yielded greater effects on weight loss than those with lower levels of adherence to recommendations [12].

The Dutch Diabetes Federation has developed a protocol for coaching individuals with IFG in a sustainable healthy lifestyle by providing information and motivational support to help them change their lifestyle. The protocol *‘Road map towards diabetes prevention’* (RM protocol) is a one-year nurse-led intervention. The present study aimed to assess the feasibility and the effectiveness of the RM protocol and the possible sustained effects of the intervention at one year follow up. Our research questions were as follows:

1. Is implementation of the RM protocol feasible in a primary care setting?
2. How effective is the protocol (12 months) and the 12-month follow-up in influencing body mass index (BMI), anthropometrics and biochemical outcomes?

## Methods

### Study population and data collection

A clustered randomized controlled trial was carried out in primary care practices in a rural area in the North-eastern region of the Netherlands. At the time of the study this region was an innovator in diabetes management, and the approach to diabetes care and prevention pioneered in the region has since become the standard for the Netherlands as a whole. Only practices that employ a practice nurse were included. In the Netherlands, practice nurses, under supervision of a general practitioner, are responsible for chronic disease management, including preventive lifestyle advice. General practices within the same primary care practice were allocated to the same study group. The number of general practitioners providing care within a primary care practice varied; in some practices only a single general practitioner provided care, whereas up to eight general practitioners provided care in larger practices. Clustering the practices was necessary, while contamination of the two care strategies might have occurred when a practice nurse carried out both the intervention and usual care within the same care practice. Consultations with a general practitioner and a practice nurse are free of charge in the Netherlands. Medications are fully reimbursed, after an initial financial contribution up to a yearly maximum of €350. Reimbursement of consultations with a dietician or physiotherapist depends on the patient’s insurance policy.

In 2010 all primary care practices (*n* = 170) in a rural part of the North-east Netherlands were invited to participate via various channels including healthcare groups, information evenings for practice nurses and direct invitation by the Dutch Diabetes Federation. Twenty-six primary care practices and 43 general practitioners were included in the study. In 2010, a computerised random number generator was used by the researchers to allocate the participating primary care practices: 12 were allocated to the intervention (IG) and 14 to the usual care group (UCG). Four practices subsequently decided not to participate, one in the IG and three in the UCG.

Participants were recruited by the general practitioner and/or practice nurse between May and November 2010 [13]. Screening techniques were allowed to vary between practices in order to follow the recruitment strategies used in daily practice as closely as possible. One of the most frequently used strategies was opportunistic screening during consultations with the general practitioner and/or practice nurse. Another approach was to select individuals systematically by age (65 years and older) and contact them by letter together with a questionnaire that provides insight into the probability of developing diabetes within five years [14]. After completing the questionnaire, those at risk were asked to make an appointment with the practice nurse. Fasting glucose measurements were taken in all screened cases. Patients were included when they were 45 years or older and newly diagnosed with IFG according to WHO criteria; (fasting glucose 6.1 to 6.9 mmol/l). As motivation is an important factor in changing lifestyle, only motivated patients were included in the study. Whether a person was sufficiently motivated to participate in the trial was judged by the practice nurse [15].

Patient exclusion criteria were: previous education on the subjects of IFG or T2DM; emotional, psychological or intellectual problems that were likely to limit their ability to comply with the protocol, and malignant or other diseases associated with a poor prognosis. The exclusion criterion ‘previous education’ was assessed by asking whether the patient had previously been informed in any way about IFG or diabetes. All participants provided a written informed consent. The study was approved by the medical ethics committee of the Isala Clinic Zwolle, the Netherlands.

During the first year IG participants received care as described in the RM protocol. In the second year they received usual care. Participants in the UCG received only usual care throughout the two-year period. General practitioners and practice nurses could therefore not be blinded to randomisation. The participants themselves were unaware of the randomisation and their particular study group.

### RM protocol

**Fig. 1:**
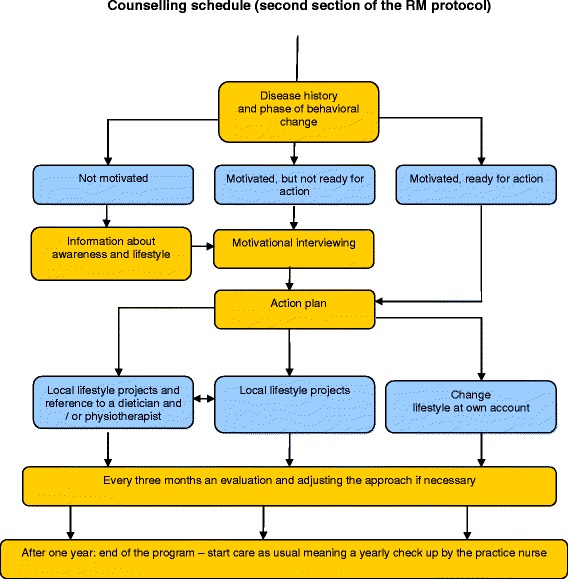
Counselling schedule (second section of the RM protocol)

Development of the RM protocol was practice-based, with the protocol focusing on stimulating individuals to adopt a healthy lifestyle. The protocol has been previously piloted to improve feasibility, with the present study based on the practical needs identified by practice nurses and general practitioners. Details of the intervention have been published previously [13]. In brief, practice nurses managing the IG were trained in the RM protocol during a single half-day session and in techniques of motivational interviewing and assessment of patient motivation over two half-day sessions. Participating subjects followed a protocol. After subjects were diagnosed with IFG (first section of the protocol) the second section of the RM protocol is applied (Fig. 1). The protocol prescribed three extra consultations in the first three months after inclusion, followed by a consultation every three months. Depending on the general practice and/or the subjects’ needs, a consultation required 10–30 minutes. During the consultation the practice nurse coached the participants by providing advice and by teaching concrete and relevant skills to help promote greater levels of physical activity and better dietary habits [13, 16]. The education and counselling received by an individual depended on their level of motivation to change their lifestyle, and on the stage of change including knowledge, pre-contemplation, contemplation, preparation, action and maintenance [16]. Participants were referred to a dietician, physiotherapist and/or local sports activities at the discretion of the practice nurse, with referral depending on the participants’ motivation and preferences.

### Usual care

According to existing guidelines from the College of General Practitioners, individuals with IFG should be tested for diabetes every year [3]. Guidelines for cardiovascular risk management advise consultation within a primary care practice every three or six months in cases with known hypertension or dyslipidaemia. Blood glucose and lipid levels should be measured on an annual basis in this patient category [17]. IFG without hypertension or dyslipidaemia commonly results in less structured cardiovascular risk management, since guidelines do not provide clear instructions for this particular patient group.

### Outcome measures

The primary outcome measure was the two-year change in body mass index (BMI). BMI was chosen as the primary outcome measure since it is objectively measurable and because we were not in a position to provide accelerometers to assess total ambulatory activity in all participants. Using levels of fasting glucose as a primary outcome measure would have required a far greater number of participants. Secondary outcome measures were body weight, waist circumference, degree of reported physical activity, total and saturated fat intake, systolic blood pressure, fasting blood glucose, total cholesterol, HDL cholesterol, triglycerides and the behavioural determinants of risk perception, knowledge and motivation. Measurements were performed, in both the IG and UCG, at baseline (T0) just after the diagnosis of IFG and before the start of the intervention, and after one (T1) and two (T2) years.

Anthropometric parameters, diabetic risk factors such as a family history of diabetes, and comorbidities including hypertension and hypercholesterolemia were measured and registered on a case report form by the practice nurse. Waist circumference and blood pressure were assessed twice on each occasion. If the results differed more than 5 %, a third measurement was taken. Biochemical parameters were determined in regional laboratories.

Participants received a questionnaire at T0, T1 and T2 from the practice nurse to be filled out at home and sent back directly to the researchers. The questionnaire at T0 comprised general background information on sex, age, ethnicity, education, household composition and occupational situation. Total and saturated fat intake were assessed using a self-reported validated questionnaire [18]. Physical activity was estimated using a short questionnaire to assess health enhancing physical activity (SQUASH) and to calculate the minutes of light, moderate and intense physical activity per week [19]. The results on the SQUASH index were plotted against the Dutch Physical Activity Norm [20]. The Dutch Physical Activity Norm states: at least 30 minutes of moderately or intense physical activity on at least five days a week (yes or no) [21]. Motivation was measured by a non-validated question: ‘to what extent are you motivated to take measures to avoid getting diabetes?’ (1 = ‘to a very high degree’ to 5 ‘not at all’).

### Process evaluation

A process evaluation was carried out in the intervention and control groups. The practice nurse recorded the number of consultations on a yearly basis at T1 and T2. After two years of follow-up a semi-structured face-to-face interview took place (by AH and MM) with the practice nurse in all practices. Interviews were taped and later summarised. The following items were discussed: the number of consultations during the first and second year of the study, the feasibility of the RM protocol, the inclusion of participants, the education and motivation of the practice nurse, the number of referrals to a dietician, physiotherapist or a local lifestyle project, and experienced the motivation and treatment possibilities of the participants.

### Statistical analysis

Based on the results of the Dutch SLIM study, the sample size was determined based on an objective to detect a decrease in BMI of 0.5 kg/m2 in the IG [9]. To adjust for clustering, an intra-cluster correlation of 0.01 was used [22]. With a power of 80 % and a two-sided alpha of 0.05, 120 people were needed in each group. Analyses were based on intention–to–treat. Possible differences between the IG and UCG at baseline were tested using unpaired t-tests or Chi-square-tests. Since both the quality and intensity of lifestyle advice is likely to be influenced by the healthcare provider, participant-related outcomes will not be fully independent but will cluster within practices. Analyses with a multilevel structure were therefore used to determine the effectiveness of the RM protocol after one and two years follow–up, applying linear and logistic multilevel analyses in SPSS 21.0 (2012.Armonk, NY: IBM Corp.) and MLwiN version 2.30 (2014 Centre for Multilevel Modelling, University of Bristol, Bristol, UK). The included levels in MlWin were: (a) the individual patient, (b) primary care practice, and (c) time. All analyses were corrected for the baseline measurements and existing differences between both groups at baseline.

## Results

**Fig. 2:**
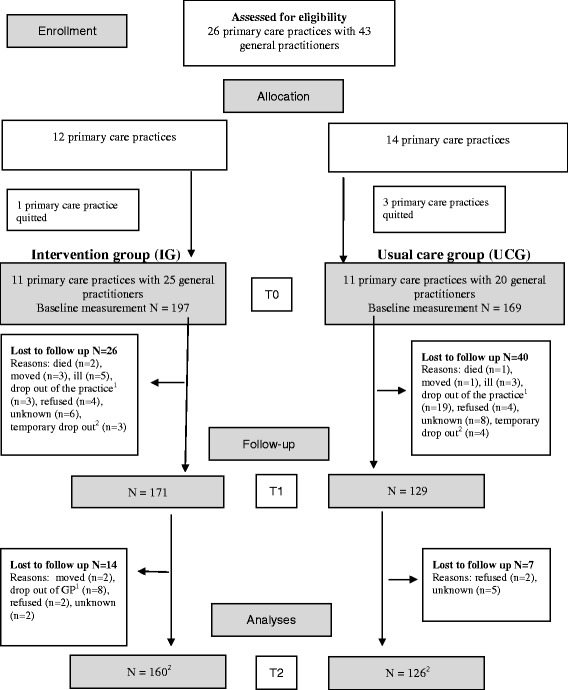
Participant flow chart and intervention compliance

A flow chart for participants is presented in Fig. 2 [23]. Baseline measurements were carried out in 366 participants. A baseline measurement and at least one follow-up measurement was available for 300 participants (IG N = 171; UCG N = 129). There were no differences between participants lost to follow-up in the IG compared to those in the UCG (data not shown). Baseline characteristics are given in Table 1 and in the second columns of Tables 2 and 3. Participants in the IG were significantly younger (62.4 vs. 65.1, *p* = 0.02) and more motivated to change their lifestyle (45.5 % vs. 30.1 %, *p* < 0.01) than those in the UCG.

**Table 1:** Baseline characteristics, patients with at least one follow-up measurement

	Intervention group N = 171 % or mean (s.d.)	Usual care group N = 129 % or mean (s.d.)
Gender		
Male	53.2	53.5
Age (range)	62.4 (9.8)*	65.1 (9.7)*
Education		
≤ Primary education	10.9	18.9
Secondary school	45.5	45.1
Selective secondary	26.1	18.0
Higher	17.6	18.0
Smoking		
Current	14.5	14.8
Former	54.2	50.8
Never	31.3	34.4
Motivated^a^		
To a very high degree	45.5	30.1*
Not at all to a high degree	54.5	69.9
Family history of diabetes		
Direct family^b^	43.7	43.3
Hypertension	48.0	43.4

*Significant difference between the intervention and control groups (*p* ≤ 0.05)

^a^‘The cut-off point was pragmatically chosen, based on the number of patients per category’

^b^Father, mother, brother or sister

**Table 2:** Changes in anthropometric and biochemical outcome measures

	T0 mean (sd)	Change T1-T0 Mean (sd)	score T2-T0 Mean (sd)	Univariate T1 β (95 % BI)	analyses^a^ T2 β (95 % BI)	MLwin^b^ T1 and T2 β (95 % BI)
BMI (kg/m^2^)						
IG (N = 168–155)	29.5 (4.8)	−0.7 (4.7)	−0.6 (4.9)	−0.16 (−0.54;0.23)	−0.98 (−2.23;0.28)	−0.21 (−0.68;0.26)
UCG (N = 117–111)	30.3 (5.1)	−0.8 (4.9)	−0.3 (5.3)			
Weight (kg)						
IG (N = 168–155)	88.4 (15.9)	−1.8 (16.2)	−1.4 (16.0)	−0.28 (−1.43;0.87)	−0.46 (−1.86;0.94)	−0.48 (−1.95;1.02)
UCG (N = 118–112)	90.3 (18.2)	−2.5 (18.1)	−1.1 (18.3)			
Waist circumference (cm)^c^						
IG (N = 165–157)	104.0 (11.7)	−2.3 (12.2)	−2.1 (12.5)	0.12 (−1.41;1.64)	−0.94 (−2.64;0.97)	−0.44 (−0.89;1.17)
UCG (N = 98–89)	104.3 (12.9)	−3.8 (13.1)	−2.2 (13.5)			
Blood glucose (mmol/L)						
IG (N = 171–154)	6.40 (0.34)	−0.23 (0.65)	−0.18 (0.68)	0.09 (−0.05; 0.24)	0.10 (−0.06;0.26)	0.05 (−0.11;0.20)
UCG (N = 118–111)	6.34 (0.46)	−0.24(0.59)	−0.25(0.62)			
Systolic blood pressure (mmHg)						
IG (N = 169–154)	140.3 (18.0)	−3.2 (14.7)	−5.4 (16.4)	−2.45 (−5.86; 0.95)	0.67 (−2.80;4.15)	−1.39 (−4.46;1.69)
UCG (N = 119–111)	141.1 (15.1)	−1.1 (14.9)	−3.1 (17.5)			
Total cholesterol (mmol/L)						
IG (N = 167–153)	5.18 (1.10)	−0.03 (1.07)	−0.12 (1.04)	−0.05 (−0.25;0.16)	−0.10 (−0.33;0.13)	−0.12 (−0.34;0.11)
UCG (N = 114–109)	5.20 (1.10)	+0.02 (1.10)	−0.03 (1.13)			
HDL cholesterol (mmol/L)						
IG (N = 167–152)	1.31 (0.31)	+0.05 (0.37)	+0.07 (0.40)	−0.04 (−0.09;0.01)	−0.01 (−0.04;0.07)	−0.03 (−0.10;0.05)
UCG (N = 115–109)	1.27 (0.31)	+0.11 (0.37)	+0.05 (0.34)			
Triglycerides (mmol/L)						
IG (N = 166–152)	1.64 (0.83)	−0.05 (0.97)	−0.013 (0.85)	−0.03 (−0.20;0.14)	−0.12 (−0.30;0.06)	−0.07 (−0.25;0.11)
UCG (N = 115–108)	1.76 (0.96)	−0.05 (0.98)	−0.04 (1.06)			

*β* Beta, *OR* odds ratio, *CI* confidence interval, *IG* intervention group, *UCG* usual care group

^a^Univariate analyses corrected for motivation and age, both on a continuous scale, and the baseline measurement

^b^Multilevel analyses corrected for motivation and age, both on a continuous scale, and the baseline measurement

^c^No data was available on waist circumference of 15 % of the participants because these patients refused this measurement

**Table 3:** Changes in physical activity, total and saturated fat intake and motivation

	T0 Mean (sd) or %	Change T1-T0 Mean (sd) or %	score T2-T0 Mean (sd) or %	Univariate T1 β (95 % BI) or OR (95 % BI)	Analyses^a^ T2 β (95 % BI) or OR (95 % BI)	MLwin^b^ T1 and T2 β (95 % BI) or OR (95 % BI)
Meets standard physical activity norm (yes)^c^						
IG (N = 166–142)	65.1	+12.1	+12.4	3.53 (1.69;7.37)**	1.22 (0.62;2.42)	1.97 (1.22;3.20)*
UCG (N = 125–116)	64.0	+4.3	+9.3			
Total fat intake^d^						
IG (N = 168–144)	18.2 (5.9)	−2.6 (5.5)	−2.8 (5.4)	−0.27 (−1.27;0.73)	−0.14 (−1.17;0.89)	−0.33 (−1.27;0.62)
UCG (N = 127–118)	18.3 (5.5)	−2.0 (5.2)	−2.6 (5.1)			
Saturated fat intake (high score)^d^						
IG (N = 168–144)	67.3	- 3.7	−6.9	1.64 (0.90;2,98)	1.42 (0.76;2.66)	1.61 (1.05;2.47)
UCG (N = 127–118)	70.1	−13.3	−13.3			

*p*-value: *P < 0.05, ** < 0.01; *β* Beta, *OR* odds ratio, *CI* confidence interval, *IG* intervention group, *UCG* usual care group

^a^Univariate analyses corrected for motivation and age, both on a continuous scale, and the baseline measurement

^b^Multilevel analyses corrected for motivation and age, both on a continuous scale, and the baseline measurement

^c^Percentages shows the number of participants meeting the Dutch Physical Activity Norm^20^

^d^A high score on fat intake is negative.

### Outcome measures

After one and two years both groups showed some reduction in BMI, body weight, waist circumference and most biochemical outcomes (Table 2). However, no significant differences were found between groups for either year.

Of the participants in the IG who did not meet the Dutch Physical Activity Norm at baseline (i.e. at least 30 minutes of moderately or intense physical activity on at least five days a week), 12.1 % did so after one and 12.4 % after two years, respectively. In the UCG these percentages were 4.3 % and 9.3 %, respectively. Multilevel analyses revealed that, compared to the UCG, the number of participants who met the norm in the IG increased significantly after one year (IG vs. UCG: OR = 1.22; 95 % CI 0.62-2.42) and over the whole study period (IG vs. UCG: OR = 1.97; 95 % CI 1.22-3.20) (Table 3). Additionally, a positive trend was seen in both groups towards diminished total fat and saturated fat intake, although no significant differences were detected between groups.

### Process evaluation

The number of consultations was significantly higher in the IG compared to the UCG during the first year (Table 4). Nevertheless, in the first year 47.9 % of the participants in the IG received fewer than the recommended five consultations prescribed in the RM protocol. In addition, 65.9 % of participants in the UCG received more than the single consultation prescribed for IFG usual care in the guidelines of the College of General Practitioners [3]. In the second year, both groups received more care than foreseen but there were no significant differences between the IG and the UCG. Analysis of interviews showed that the main motivation of practice nurses to provide more care to the UCG than initially foreseen was as a response to the needs and wishes of the participants, thus striving for customized care. A practice nurse for the UCG commented: *‘We consider a patient’s risk factors and their personality. We do not really follow protocol, but rather tailor a plan for each individual patient.*

**Table 4:** Mean number of consultations in the IG and UCG

Number of consultations	IG		UCG	
	N	(%)	N	(%)
during the trial				
3 or less	33	(19.5)	44	(34.1)**
4	48	(28.4)	18	(14.0)
5 or more	88	(52.1)	67	(51.9)
N	169^a^		129	
in the year of follow-up				
0	9	(5.7)	8	(6.8)
1	27	(17.2)	21	(17.8)
2–3	71	(45.3)	43	(35.9)
4 or more	50	(31.8)	46	(39.0)
N	157		118	

*p*-value: ** < 0.01;

^a^The number of consultations is lacking for two participants

Additionally, as 43 % of participants in the UCG had hypertension this necessitated a higher frequency of check-ups than simply one a year. Compared to the UCG, participants in the IG were more often referred to a dietician or physiotherapist in the first study year (IG 22 % vs. UCG 13 %; *p* = 0.05).

Practice nurses expressed positive opinions regarding the RM protocol and its effects on participants. Achieving the agreed level of consultations appeared to be less practicable, since almost half of the participants did not receive the prescribed four to five consultations. Practice nurses mentioned that participants found the number of consultations rather high, especially since they did not have a serious perception of risk or a sense of a relevant disease burden. Practice nurses in the IG mentioned: ‘*Patients consider it unnecessary to invest time in visits. They claim that they don’t feel sick and that ‘people with IFG are generally young and don’t have time for frequent visits to a practice nurse’.*

Practice nurses in the IG were slightly more positive about their skills in managing people with IFG and stimulating dietary changes and degree of physical activity than practice nurses in the UCG. Practice nurses for both the intervention and control groups judged the participants’ feelings of responsibility and motivation as moderate at best.

A practice nurse stated: *‘After a good explanation patients are prepared to change their lifestyle as they leave the room, but at home this positive attitude and motivation disappears.’* Additionally, practice nurses mentioned that the perceived effectiveness of the RM protocol on lifestyle and weight loss was larger for intrinsically motivated patients.

## Discussion

A pragmatic, nurse-led protocol that aimed to achieve lifestyle changes in subjects with IFG was not as effective as originally hoped. Although both groups showed a reduction in BMI directly after the trial and at 12 months follow-up, no significant differences between the IG and UCG were found. Comparable effects were also found on secondary outcome measures such as fat intake and several anthropometric and biochemical outcomes. A significant improvement in the number of individuals meeting the standard physical activity norm was found, however. The trial was conducted in normal practices and outcomes might thus be related to differences in consultation rates between the IG and UCG, a non-compliance factor possibly related to participants’ sense of a low disease burden. Another factor might have been the limited capacity of practice nurses (with minimal training) to motivate lifestyle changes in participants [24]. This lack of motivation, which emerged clearly during process evaluation, was probably a major contributory factor to the lack of effect of the RM protocol. This conclusion also emerged from a study of patients with a recent diagnosis of T2DM, which reported that participants failed to take their condition seriously and postponed lifestyle changes until diabetes-related complications appeared [25].

An increase in reported physical activity is an important outcome in the context of prevention, since only a small improvement in physical activity reduces the risk of T2DM [26] and lowers all-cause morbidity and mortality risk in men and women [27]. Since many individuals with IFG also have (a propensity to) cardiovascular diseases, physical activity is an important lifestyle change [2]. In contrast to our findings, studies that implemented intensive programs found significant and relevant reductions in BMI, body weight and waist circumferences in the intervention group [10, 12, 28, 29]. However, and in agreement with our study, they were unable to detect effects of the intervention on blood glucose, blood pressure, cholesterol or triglyceride levels [10, 11, 29–32].

Participants in the IG received less care than the four to five consultations in the first year prescribed by the RM protocol. Additionally, only a quarter of the participants were referred to a dietician or physiotherapist. This low number of referrals was at least partly due to the lower reimbursement of dietician or physiotherapist consultation costs, which was unexpectedly introduced by health insurance companies at the beginning of the study. However, it is also important to consider the possibility that the low referral rate might reflect the poor motivation and willingness of participants to change their lifestyle. In fact, practice nurses in the IG mentioned that a sizeable number of participants with IFG did not use the extra’ consultations (free of charge) offered to them, citing a lack of any sense of disease burden amongst participants. This finding is in line with results from a study of individuals with a recent diagnosis of T2DM but without complications [25], and was confirmed by our data on the decrease in motivation in the IG after one and two years (data not shown). Additionally, those receiving more consultations were probably more motivated. A per protocol analysis that included only those in the IG with at least four and in the UC only those with a maximum of three consultations, revealed an intervention effect on reported physical activity norms and total fat intake (data not shown).

Based on Dutch standards, the number of consultations in the UCG was higher than expected [17]. Two explanations might explain this unexpected increase: The un-blinded design may have allowed both practice nurses and participants to discover IFG status, influencing perceived risk of T2DM and encouraging the nurse to deliver extra care. Additionally, almost half of all participants had hypertension, which is an indication for extra consultations and coaching according to the guidelines for cardiovascular risk management [3, 17]. Awareness of hypertension after inclusion in the study might have led to more visits than originally envisioned [17].

One important element of the RM protocol was motivational interviewing. Recent studies have indicated that training of practice nurses in motivational interviewing does not produce the expected changes in lifestyle related to diet and physical activity in participants with T2DM [33]. The same might also hold true for subjects with IFG.

### Strengths and weakness

Important strengths of the study included a setting within working practices and the relatively low number of dropouts (19 % after one and 24 % after two years) compared to other studies (with dropout rates from 11 to 59 % after one year) [10, 11]. As general practices were free to select participants, a weakness might be a possible selection bias [11]. Additionally, to avoid interactions between practice nurses and participants in the IG and UCG, randomization took place at the level of group practices. Despite this, practice nurses in the usual care arm were aware of the study, and one cannot exclude the possibility that at least some of them were aware of interventional locations and details, despite our best efforts to isolate nurses in the respective study arms as regards education and trial information. Another possible weakness of the study is that participating practices might have been more motivated and interested in lifestyle counselling than other non-participating practices. This cannot be prevented in a real life setting however. Another important point is that certain outcome measures such as body weight, waist circumference and blood pressure were measured by practice nurses. This might have allowed bias, especially since practice nurses were not blinded to treatment allocation. Furthermore, although motivation was an important aspect of the study, this was assessed using a non-validated single item question. On the other hand, as this was not an outcome measure of the study it did not influence our results and conclusions.

## Conclusions

A relatively simple lifestyle program in individuals with impaired fasting glucose resulted in a significant improvement in reported physical activity, but not in BMI and anthropometric parameters. Despite the fact that the program is less intensive than most other programs, for a substantial proportion of the studied population and for some practice nurses it appeared to be too intensive. This attitude might have been related to a lack of motivation amongst participants and to the fact that subjects with IFG do not experience a disease burden [25]. In line with other recent research in this field, we suggest that illness perceptions should be taken into account when drafting a protocol such as the RM protocol, and that a tailored consultation scheme should be included.

## Abbreviations

IG: Intervention group
UCG: Usual care group
RM: protocol ‘Road map towards diabetes prevention’
IFG: impaired fasting glucose
